# Effects of Active Chronic Cigarette-Smoke Exposure on Circulating Fibrocytes

**DOI:** 10.1007/s00408-024-00720-3

**Published:** 2024-06-27

**Authors:** Faheem Khan, Eoin P. Judge, Jeeban P. Das, Daniel White, Carolyn Ingram, Michael P. Keane, Marcus W. Butler

**Affiliations:** 1 St Vincent’s University Hospital, Elm Park, Dublin 4 Ireland; 2 University College Dublin, Belfield, Dublin 4 Ireland; 3 Education & Research Centre, St Vincent’s University Hospital, Elm Park, Dublin 4 Ireland

**Keywords:** Circulating fibrocyte, Smoking, Chronic obstructive pulmonary disease, Idiopathic pulmonary fibrosis

## Abstract

**Purpose:**

This study aimed to evaluate the hypothesis that active smoking impacts upon mediators and abundance of circulating fibrocyte cells in smoking-related disease characterised by fibrosis.

**Methods:**

Flow cytometry and enzyme-linked immunosorbent assays were used to investigate blood from five patient groups: healthy never-smokers, healthy current smokers, stable chronic obstructive pulmonary disease (COPD) active smokers, idiopathic pulmonary fibrosis (IPF) never-smokers, and IPF active smokers.

**Results:**

A significant inverse dose–response relationship was observed in healthy smokers among cumulative smoking burden (pack-years) and fibrocyte abundance (*p* = 0.006, *r* = −0.86). Among serum profibrotic fibrocyte chemokines measured, CCL18 rose significantly alongside fibrocyte numbers in all five subject groups, while having an inverse dose–response relationship with pack-year burden in healthy smokers (*p* = 0.003, *r* = −0.89). In IPF, CCL2 rose in direct proportion to fibrocyte abundance irrespective of smoking status but had lower serum levels in those currently smoking (*p* =  < 0.001). For the study population, CXCL12 was decreased in pooled current smokers versus never-smokers (*p* = 0.03).

**Conclusion:**

The suppressive effect of current, as distinct from former, chronic smoking on circulating fibrocyte abundance in healthy smokers, and modulation of regulatory chemokine levels by active smoking may have implications for future studies of fibrocytes in smoking-related lung diseases as a potential confounding variable.

**Supplementary Information:**

The online version contains supplementary material available at 10.1007/s00408-024-00720-3.

## Introduction

Fibrocytes are fibroblast-like peripheral blood cells that migrate to the regions of tissue injury [1]. These hematopoietic cells comprise 0.1 – 0.5 percent of the circulating leukocyte population and are variably defined by the expression of type I collagen (Col-1), CXCR4, CD11b, CD13, CD34, CR45, MHC II, and CD86 [2–5]. Fibrocytes constitute bone marrow precursor cells believed to transform into fibroblasts or myofibroblasts, under the influence of different molecules including cytokines, growth factors, and components of the extracellular matrix (ECM) [6–12].

Chronic obstructive pulmonary disease (COPD) and idiopathic pulmonary fibrosis (IPF) are two chronic, severe smoking-related fibrogenic lung diseases. The role of circulating fibrocytes in the pathogenesis of IPF is unclear, though several studies have implicated the CXCL12-CXCR4 chemokine axis in murine models and enhanced expression of CXCL12 in human lung tissue and plasma [4, 13]. An unanswered question concerns the origin of the peribronchial fibrogenesis observed in COPD airway walls [14].

Circulating fibrocyte levels are upregulated in stable IPF and further so in acute exacerbations of IPF. There are limited data on fibrocyte biology in COPD, though smoking/COPD have a depressive effect on bone marrow-derived precursor cell abundance in general, within which fibrocytes could be differentially affected [15]. Furthermore, opposing effects of cigarette smoke extract and nicotine on bone marrow-derived mesenchymal stem-cell (MSC) function and their progeny [16–19]. In that context, it was hypothesised in the present study that smoking burden impacts circulating fibrocyte levels and in a dose-dependent manner. Furthermore, it was postulated that smoking status may discordantly influence the abundance of circulating fibrocytes in stable IPF, COPD smokers, ostensibly “healthy” smokers and healthy non-smokers. Finally, it was hypothesised that relevant blood markers of fibrocyte chemotaxis/signalling would be differentially impacted in smoking-related phenotypes.

## Materials and Methods

### Study Population

Seventeen age-matched healthy volunteers, i.e. nine healthy non-smokers (H–NS), and eight healthy smokers (H–S) were studied as controls. Ten actively smoking patients diagnosed with COPD (COPD–S) as per standard Global Initiative for Obstructive Lung Disease (GOLD) criteria [20], 7 IPF non-smokers, and 4 current smokers with IPF (IPF–NS and IPF–S, respectively) as defined per standard ATS/ERS diagnostic criteria [21] were recruited from respiratory outpatient clinics, the acute medical assessment unit and emergency department of the host institution, i.e. St Vincent’s University Hospital, Dublin, Ireland. The research protocol was approved by the host institute’s research and ethics committee and informed consent was obtained (For inclusion and exclusion criteria, see supplementary file).

Self-reported smoking status was confirmed objectively by urinary tobacco metabolite—cotinine assays using commercial kits (GFC Diagnostics, Oxfordshire, UK) [22].

### Experimental Techniques

Peripheral blood mononuclear cells (PBMC) and serum were isolated from peripheral blood of all subjects using a gradient with ficoll-paque. 1 ml aliquots of cells were placed at −80 °C in a freezer overnight before transferring to long-term liquid nitrogen storage pending their use for flow cytometry. Fibrocytes were identified as cells positive for anti-CD45, Collagen-1, and CXCR4 antibodies and fibrocyte concentrations were measured relative to total leucocyte counts and as absolute numbers (For details, see supplementary file). The serum sample from each subject was stored at −80 °C until chemokine/cytokine analysis. The concentrations of CXCL12, CCL2, CCL18, interleukin 12 (IL–12), interferon Gamma (IFN–*γ*), and transforming growth factor beta 1 (TGF–*β*1) in the serum were determined by enzyme-linked immunosorbent assays (ELISA) using commercial kits according to the manufacturer’s instructions (For manufacturer details, see supplementary file).

Statistical analyses were conducted using SPSS version 29. To account for small sample sizes and non-normally distributed outcomes, nonparametric tests were used to compare independent samples. Categorical variables were compared between phenotype groups using Fisher’s exact test and continuous variables with the Mann–Whitney *U* test (if two groups compared) or the Kruskal–Wallis *H* test (if three or more groups compared). Spearman’s rank order correlation (*ρ*) was used to assess the relation between continuous and/or discrete variables. To account for family-wise error rate associated with multiple comparisons, a Bonferroni correction was applied, and the significance threshold was set at *p* < 0.01. (for details see supplementary file).

## Results

All individuals in each study group were matched for gender distribution (*p* = 0.56) and age (*p* = 0.09). The subjects in all three current-smoker groups were matched for their respective smoking pack-year history (*p* = 0.69). Only the ratio of FEV1 to FVC (*H*(2) = 10.85; *p* = 0.004) and urine cotinine levels (*H*(4) = 32.21; *p* < 0.001)) differed significantly across the phenotype groups, in keeping with the expected airflow obstruction in COPD-S subjects and confirmed smoking status (Table 1).

**Table 1:** Baseline demographics of the study population and biologic samples*

Variables	H–NS †	H–S ‡	COPD–S §	IPF–NS ||	IPF–S **	*p*-value
N	9	8	10	7	4	
Age (years)	63.7 ± 4	61.3 ± 2.8	69.9 ± 3.5	74.1 ± 3.1	70.5 ± 4.3	H(4) = 7.80; *p* = 0.10
Sex (male/female)	6/3	3/5	4/6	3/4	3/1	Fisher-exact *p* = 0.60
Smoking status						
Current / Ex/ non-smoker	0/0/9	8/0/0	10/0/0	0/0/7	4/0/0	Fisher-exact *p* < 0.001
Smoking history (pack years)	NA	39.7 ± 6.7	38.2 ± 2.9	NA	32.5 ± 5.9	H(2) = 1.28; *p* = 0.53
Urine cotinine level (0–4) ††	0 ± 0	1.87 ± 0.22	2.6 ± 0.22	0 ± 0	2.75 ± 0.25	H(4) = 32.21; *p* < 0.001
PFT parameters ‡‡						
FEV1%	N/A	N/A	57.8 ± 9.7	77.7 ± 7.2	97.5** ± **7.9	H(2) = 7.79; *p* = 0.02
FVC %	N/A	N/A	78 ± 12.2	72.1 ± 7.3	103.5 ± 8.2	H(2) = 5.25; *p* = 0.07
FEV1/FVC %	N/A	N/A	60.9 ± 6.8	85.8 ± 2.1	74.1 ± 4.8	H(2) = 10.85; *p* = 0.004
DLCO %	N/A	N/A	71.5 ± 3.5	50.2 ± 1.9	51.5 ± 7.6	H(2) = 3.46; *p* = 0.18
DLCO/VA%	N/A	N/A	83 ± 1	86.2 ± 1.3	70 ± 14.4	H(2) = 2.65; *p* = 0.27

^*^Continuous data is presented as mean ± standard error of mean. Kruskal–Wallis H Test statistics and *p*-values reported for comparisons of continuous/discrete variables across phenotypes. Fisher-exact *p*-values reported for comparisons of categorical variables. A conservative Bonferroni-adjusted *p*-value was used to determine statistical significance and the significance threshold set at *p* < 0.01

^**†**^H–NS, healthy non-smokers

^‡^H–S, healthy smokers

^§^COPD–S, chronic obstructive pulmonary disease–smokers

^||^IPF–NS, idiopathic pulmonary fibrosis–non-smokers

^**^IPF–S, idiopathic pulmonary fibrosis–smokers

^†^^**†**^Urine cotinine levels as a semi quantitative test score 0–4 where 0 = non-smoker, 1 = light smoker, 2 = moderate smoker, 3 = heavy smoker, 4 = very heavy smoker

^**‡‡**^PFT, pulmonary function test. PFT parameters are given as percent of predicted value with the exception of FEV1/FVC, and DLCO/VA which is reported as % observed; FVC—forced vital capacity, FEV1—forced expiratory volume in 1 s, DLCO—diffusing capacity of the lung for carbon monoxide, and VA–alveolar volume

^§§^ns, not significant

**Fig. 1:**
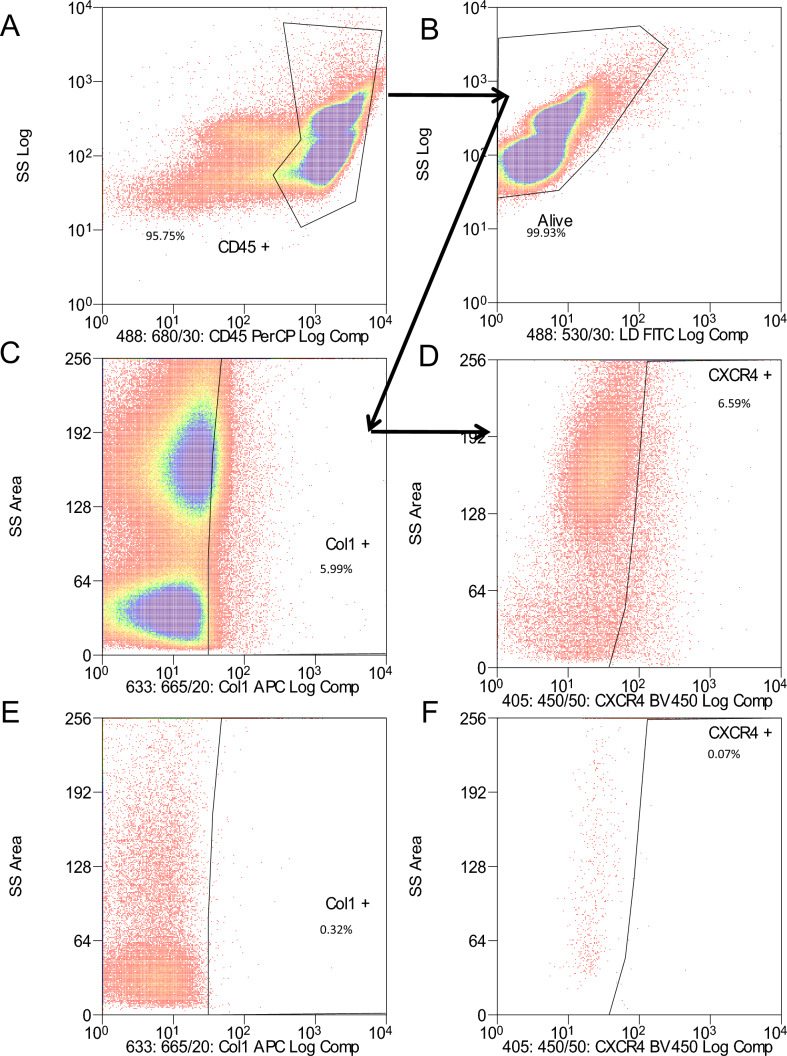
Expression of CD45, collagen I (Col I), and CXCR4 in peripheral circulating leucocytes—Representative flow cytometric analysis from a patient with COPD. **A** The total cell population was analysed for PerCP stained CD45 expression. **B** This gate was then applied to the CD45 positive cells which are live, i.e. 99.9%. **C** Live CD45 positive cell population was further evaluated for APC-stained Col I positive cells. **D** Live CD45, Col-I positive cell population was further gated down to BV450 stained CXCR4 positive cells, i.e. 3776 events of 957,460 CD45+ cells are alive Col1+ and CXCR4+ (0.39%) called fibrocytes. **E** The isotope control for APC-stained Col I cells. **F** The isotype control for BV450 stained CXCR4 cells

**Fig. 2:**
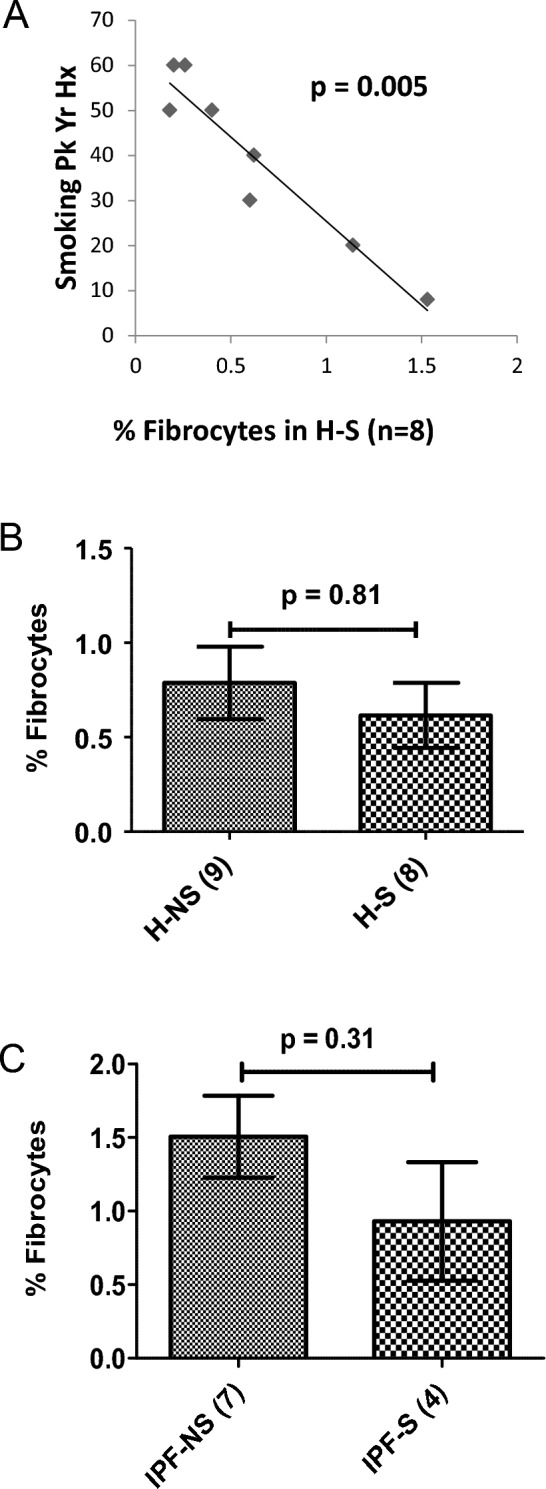
Effect of chronic smoke exposure on circulating fibrocyte abundance. **A** Relationship between cumulative burden of tobacco (expressed as cigarette pack-years) and fibrocyte abundance (% of total circulating leukocytes) in healthy smokers. **B** Prevalence of fibrocytes (% of total circulating leukocytes) in healthy subjects assessed by smoking status. **C** Prevalence of fibrocytes (% of total circulating leukocytes) in IPF non-smokers and IPF smokers

Fibrocytes were quantified by flow cytometry using the gating strategy as shown in Fig. 1 in all subjects. Given that the central hypothesis of the current study was that smoking impacts upon circulating fibrocyte levels, the relative prevalence of these cells was analysed as a function of cumulative smoking burden, initially in the absence of clinical disease. Among these H–S subjects, the analysis revealed a significant negative correlation or an inverse dose–response relationship between the smoking pack-year history and percentages of circulating fibrocytes (Spearman’s *ρ* = -0.868, 99% CI (2-tailed) −0.987 to −0.136; *p* = 0.005, Fig. 2A). Smoking pack-year was noted to vary from as low as 8 pack year to as high as 60. Despite the low number of subjects studied, the inversely proportional relationship between fibrocyte level and corresponding pack-year value did not appear to be driven by one or two outlying values, and therefore was felt to be consistent with a tight relationship of cumulative smoking burden to circulating fibrocyte level in ostensibly “healthy” smokers. The next analysis was to assess if this inverse dose–response would lead to the expected finding of fewer fibrocytes in healthy smokers than in non-smokers. There was a numerically lower mean percentage of circulating fibrocytes in the H–S group (0.61 ± 0.17%) as compared with the H-NS group (0.78 ± 0.19%) but this difference was not statistically significant (*U* = 33.5, *p* = 0.81, Fig. 2B). To see if current smoker status might account for a concordant depressive effect in another phenotypic context, the prevalence of circulating fibrocytes was next assessed against an IPF background.

The data once more revealed a numerically lower mean level of circulating fibrocytes in IPF smokers compared to non-smoker IPF subjects which did not attain statistical significance (*U* = 8.0, *p* = 0.31, Fig. 2C), likely impacted by the very small number (*n* = 4) of IPF current smokers that could be found for this study.

**Fig. 3:**
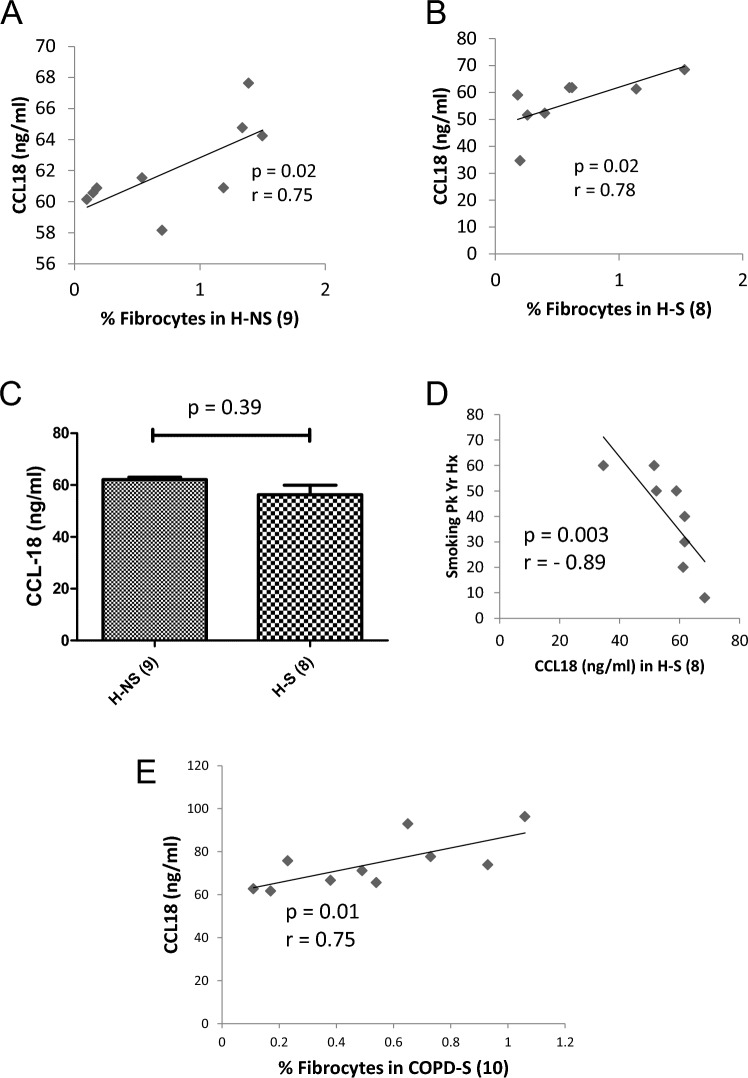
Role of Serum CCL18 in smoking-induced fibrocyte regulation in healthy subjects. **A** Relationship between serum CCL18 and circulating fibrocytes as a percentage of total leukocytes among healthy non-smokers. **B** Relationship between serum CCL18 and circulating fibrocytes as a percentage of total leukocytes among healthy smokers. **C** Serum CCL18 levels in healthy non-smokers versus healthy smokers. **D** Relationship of serum CCL18 levels to cigarette pack-year burden in the healthy smoker group. **E** Relationship between serum CCL18 and circulating fibrocytes as a percentage of total leukocytes among COPD smokers

Considering the observed inverse dose–response relationship of smoking burden and fibrocyte level, an exploratory analysis was conducted of potential associations among plausible chemokine markers of circulating fibrocyte activation in serum and smoking status in healthy individuals. CCL18, derived from alveolar macrophages (a cell type exposed directly to smoke) is a known mediator of fibrogenesis implicated in the smoking-related lung disease IPF, and in the ILD associated with systemic sclerosis, another disease where fibrocytes play a role [23]. The data indicated that there was a statistically insignificant, directly proportional relationship among CCL18 levels and fibrocyte levels, consistent with possible candidate status for CCL18 in fibrocyte regulation, observed in the H–NS (*ρ* = 0.75, 99% CI 0.086–1.0; *p* = 0.02, Fig. 3A) and H–S cohorts (*ρ* = 0.78, 99% CI 0.0151–1.0, *p* = 0.02, Fig. 3B). The mean levels of CCL18 were similar in both groups (*U* = 27.0, *p* = 0.39, Fig. 3C). There was a statistically significant inverse relationship among pack-year value and CCL18 level in the H–S group, consistent with (though not proving) the potential for CCL18 to be involved in mediating the inverse dose–response relationship observed among circulating fibrocyte levels and the burden of chronic smoke exposure (*ρ* = 0.89, 99% CI 0.123–1.0; *p* = 0.003, Fig. 3D). The same directly proportional relationship of serum CCL18 with fibrocyte abundance seen in each healthy group was observed in the larger group of COPD smokers (*p* = 0.75, 99% CI −0.086 to 1.0; *p* = 0.01, Fig. 3E).

**Fig. 4:**
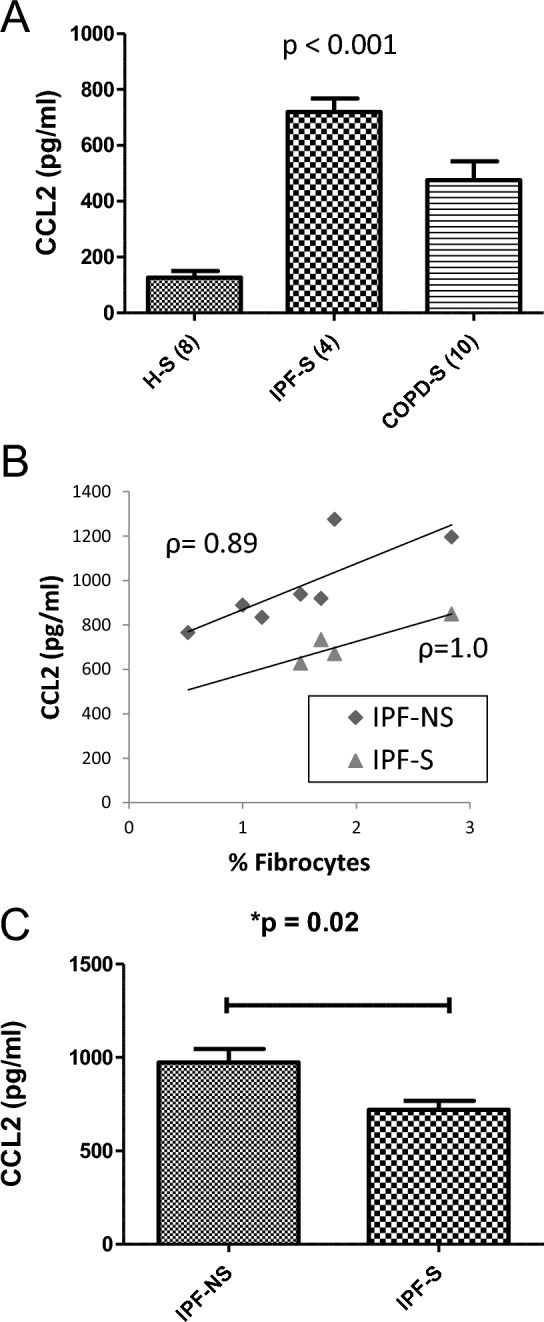
Association of serum CCL2 with circulating fibrocyte abundance and smoking status in idiopathic pulmonary fibrosis. **A** Concentration of serum CCL2 among healthy smokers, IPF smokers, and COPD smokers. **B** Relationship of serum CCL2 to circulating fibrocyte prevalence among IPF non-smokers and IPF smokers. **C** Concentration of serum CCL2 in IPF non-smokers and IPF smokers

The chemokine CCL2/MCP-1, implicated in fibrocyte recruitment via its cognate receptors CCR2 and CCR4 has been shown in IPF to be at elevated concentrations in serum and BALF [24]. The data herein confirmed a significantly higher serum CCL2 level in IPF smoker subjects compared to healthy smokers and compared to COPD smokers (*H*(2) = 16.476, *p* < 0.001, Fig. 4A). There was a direct correlation of serum CCL2 levels and percent prevalence of circulating fibrocytes among IPF non-smokers (*ρ* = 0.89, Fig. 4B) and among IPF smokers (*ρ* = 1.0, Fig. 4B).

We next sought to extend these findings by analysing the impact of smoking status in IPF on the enhanced level of CCL2 production. The data showed a trend towards a significant reduction in serum CCL2 levels in IPF smokers versus IPF non-smokers, consistent with a possible depressive effect of current smoking on a fibrocyte-associated profibrotic chemokine in IPF although the finding needs to be interpreted with caution in view of the low numbers of IPF smokers (*U* = 2.0, *p* = 0.02, Fig. 4C).

**Fig. 5:**
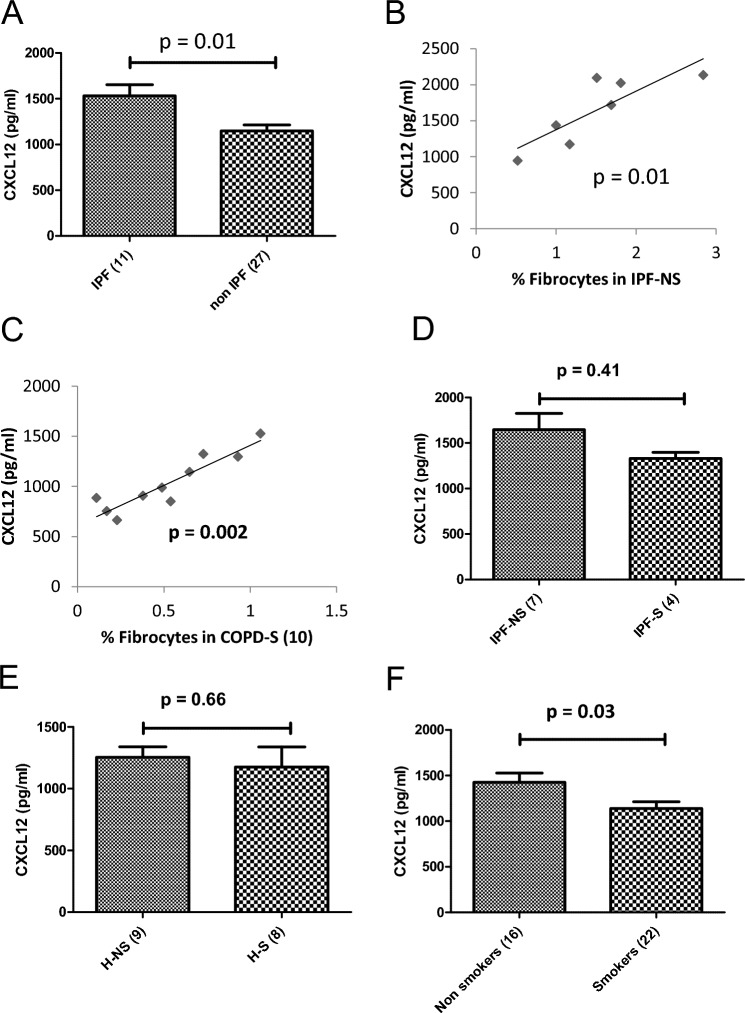
Role of Serum CXCL12 in smoking-induced fibrocyte regulation in healthy subjects, IPF and COPD patients. **A** Concentration of serum CXCL12 in IPF and non-IPF groups. **B** Relationship between serum CXCL12 and circulating fibrocytes in IPF-Non Smokers. **C** Relationship between serum CXCL12 and circulating fibrocytes in COPD smokers. **D** Concentration of serum CXCL12 among IPF non-smokers VS IPF smokers. **E** Concentration of serum CXCL12 in healthy non-smokers and healthy smokers. **F** Concentration of serum CXCL12 in all smokers VS all non-smokers

CXCL12 has attracted interest as the ligand interacting with CXCR4 in the purported migration of circulating fibrocytes to sites of injury, such as has been postulated to occur in IPF and COPD [4, 25]. Others have shown higher blood concentrations of CXCL12 in IPF, with levels directly related to levels of circulating fibrocytes [13]. In the present study, there was a significantly higher level of serum CXCL12 observed in the IPF group versus the non-IPF cohorts (*U* = 71, *p* = 0.01, Fig. 5A). Additionally, in IPF never-smokers the directly proportional relationship in blood of CXCL12 levels and fibrocyte percent prevalence was demonstrated (Spearman’s *ρ* = -0.86, 99% CI −0.677 to 1.0; *p* = 0.01, Fig. 5B) with a weaker, similar relationship observed in the small number of IPF smokers (data not shown). The same relationship was observed in the COPD smoker group (Spearman’s *ρ* = 0.84, 99% CI 0.073–1.0; *p* = 0.002, Fig. 5C). To extend upon these findings and link them back to the central hypothesis regarding how smoking impacts fibrocyte biology, CXCL12 levels were analysed by smoking status. While the observations of concordant, numerically reduced mean levels of serum CXCL12 in the smoker as opposed to non-smoker subgroups of IPF subjects (Fig. 5D) and healthy subjects (Fig. 5E) were not statistically significant, by pooling all non-smokers (healthy and IPF) compared to all pooled smokers (healthy, IPF, and COPD), there was evidence to suggest a lowering of CXCL12 levels in the current smokers (*U* = 104, *p* = 0.033, Fig. 5F).

## Discussion

In the present study, data was gathered on the influence of chronic tobacco smoke consumption on measurable features of circulating blood fibrocytes in otherwise healthy controls and the smoking-related fibrogenic lung disease states of IPF and COPD. For the first time to our understanding, a significant inverse association of cumulative self-reported tobacco burden and numbers of circulating fibrocytes in otherwise healthy smokers was seen, suggesting a depressive effect of chronic smoking on fibrocyte abundance in the absence of clinical disease. The numerically lower mean abundance of fibrocytes observed in healthy smokers versus healthy non-smokers in the current work was not significantly different, which we postulate is due to underpowering. The direction of the observed inverse dose–response relationship among circulating fibrocytes and cigarette pack-year (lowest cell abundance in longest-smoking individuals) is of central importance as it is incompatible with an inference that merely advancing age explains the higher fibrocyte levels in disease-free smokers, even though increasing age is well described as being directly associated with higher circulating fibrocyte levels [23, 26]. Although underpowered, the numerically lower mean number of fibrocytes for current smokers was concordantly seen where each of the two smoker phenotypic groups was compared with their non-smoker group (H–S vs H–NS, and separately for IPF–S vs IPF–NS). The largest, published, relevant comparator dataset we could find had mixed smoking status histories (current/former/never smoker) within each of two cohorts; COPD and age-matched controls, but interestingly, the group enriched with current smokers (COPD) had a numerically lower mean number of CD45^+^/Col1^+^ circulating fibrocytes versus the control group who were enriched for never-smokers, a result concordant with the current study findings, and deserving of investigation in an appropriately powered study [25]. No published studies have compared circulating fibrocyte proportions in COPD versus IPF. IPF has a far greater tissue burden of fibrotic lesions in the pulmonary parenchyma versus the comparatively modest peribronchial fibrosis observed in COPD pathologic specimens [14]. The current study showed concordant relationships for observed pro-fibrotic chemokines/ligands having positive correlation with circulating fibrocyte abundance irrespective of whether the phenotype was COPD or IPF. The fact that smoking has been shown to exert a depressive effect in general on bone marrow-derived progenitor cells (which could include fibrocytes) is in keeping with the current study which observed a reduction by active smoking of the elevated blood fibrocyte levels otherwise found in IPF, a depressive effect of smoking on fibrocyte abundance as was similarly observed for healthy subjects [15].

Little is currently known about the role of chemokines in any potential trafficking of fibrocytes in COPD. Tissue fibrocytes have been shown to be present at greater density in (mainly former smoker) COPD airways than in controls, and correlated with blood fibrocytes [27]. The chemokine CCL18 has some similarities with TGF-β in that it up-regulates collagen production by lung fibroblasts. To our knowledge, the current study is the first to show an association of serum CCL18 levels with increasing fibrocyte abundance in COPD, and has relevance in light of published data that emerged in the literature after the current study was undertaken, showing recruitment of blood fibrocytes during acute exacerbations of COPD, linked to worsening of clinically important endpoints including lung function and mortality [25]. In keeping with the hypothesis that smoking downregulates circulating fibrocytes, the pro-fibrotic chemokine CCL18 had levels that were inversely proportional to cumulative smoking burden in healthy smokers.

The mechanistic and prognostic role of fibrocytes is more established in IPF, a disease characterised by marked pulmonary parenchymal fibrosis and where circulating fibrocytes are strongly upregulated versus controls, and more so in acute IPF exacerbations [4, 13, 28]. Importantly, previous investigators have used human control blood samples to demonstrate in vitro a stimulatory effect of CCL2 exposure on CCR2 receptors expressed on fibrocytes in these samples resulting in proliferation of fibrocytes, migration, and differentiation into a myofibroblast phenotype [29]. Data from Ekert and co-workers supports a mechanism for the CCL2/CCR2 axis in fibrocyte pathobiology and predicts a linear relationship in blood among CCL2 and fibrocyte levels in IPF but was only tested in blood from BALB/c mice / healthy human donors and not tested in IPF subjects. To the best of our knowledge, the current data provides the first direct evidence of such a relationship in IPF subjects. There was a statistically significant direct correlation in blood of IPF non-smokers among CCL2 levels and percent prevalence of circulating fibrocytes providing external validation to the relevant work of Ekert et al. In IPF, we postulate that in a higher powered study where current smokers with IPF may have lower levels of circulating fibrocytes, CCL2 may, in part, mediate this effect based on the significant reduction in CCL2 levels observed in the current work in IPF smokers versus their non-smoker counterparts.

CXCL12 is well-implicated in fibrocyte trafficking in fibrotic lung disease [4, 13, 25]. The current study confirmed the previously reported higher levels of CXCL12 in IPF, an association of CXCL12 serum levels with fibrocyte levels in IPF and separately in COPD and showed for the first time this association in a pure population of current smokers with COPD [25]. CXCL12 is postulated herein to play a role in the hypothesised down-regulation of fibrocyte abundance in current smokers, based on the present study’s finding of significantly downregulated CXCL12 in a pooled analysis of all smokers compared to all non-smokers.

Taken as a whole, the findings are consistent with the hypothesis that chronic smoke exposure may lead to a reduction in circulating fibrocytes, bone marrow-derived cells, and that in IPF, this putative effect may be mediated by the CCL2 axis and likely also by the CXCL12 axis. The present study has important limitations however, chief of which is the low number of samples studied, and future studies would need greater sample size to generate more reliable results. A particular limitation of the study was the low numbers of IPF smokers, with a resultant failure to show statistical significance for the numerically lower mean number of fibrocytes in IPF smokers, likely due to underpowering rather than no effect of smoke on IPF fibrocytes in the authors’ view. In neither the analyses of IPF nor healthy subjects were numerically higher mean fibrocyte numbers observed in the smoker subgroups, and others have shown a reduction in bone marrow-derived cells in chronic smokers, that rebounds with smoking cessation [15, 30, 31]. The abrupt rise of circulating progenitor cells upon smoking cessation may explain the reported lack of difference in circulating fibrocyte levels in control subjects parsed by ever-smoking status (current/former smokers combined as one group versus never-smokers) in a larger study than the current one [31]. Proof of the present hypothesis will require higher numbers of subjects than were recruited herein. Future work examining how smoking affects circulating fibrocytes could evaluate isolated cell populations to measure gene expression and translation profiles in addition to functional assays that include an evaluation of chemotactic alterations.

## Conclusion

Bone marrow-derived circulating progenitor cells called fibrocytes are implicated in causation/prognosis of prevalent smoking-related lung diseases but little is known about the impact of ongoing chronic smoking on these cells. The present data shows a significant suppressive effect of ongoing cumulative smoking burden on the abundance of these cells in a dose-responsive manner in preclinical smoke-exposed subjects that may confound interpretation of data in studies of circulating fibrocytes where current as opposed to former smoking status is uncontrolled for. Larger studies are needed in this area to better understand the impact of smoking on fibrocytes in health and disease.

### Supplementary Information

Below is the link to the electronic supplementary material.

Supplementary file1 (DOCX 17 kb)

## Data Availability

No datasets were generated or analysed during the current study.
