# Supplementary material for: Effects of Active Chronic Cigarette-Smoke Exposure on Circulating Fibrocytes
Source: Lung. 2024 Jun 27;202(4):431–40. doi: 10.1007/s00408-024-00720-3 (PMC11272705; doi:10.1007/s00408-024-00720-3)
Supplement: Supplementary file 1 — Supplementary file1 (DOCX 17 kb) [file 408_2024_720_MOESM1_ESM.docx]

**Supplementary File**

1. **Flow cytometry**

PBMCs were prepared at a concentration of 1 x 10^6^ in 300 μl of fluorescence-activated cell sorter buffer (BD Biosciences, San Jose, CA). The cell surface antigens were stained with anti-CD45 and CXCR4 (CD45-PerCP, CXCR4-BV 421; Biolegends, San Diego, CA, USA). Then cells were fixed and permeabilized with Triton-X – 0.1% (93443, Sigma-Aldrich, Wicklow Ireland) for intracellular staining and were incubated with a specific monoclonal antibody for collagen I (anti-collagen type1-APC; Rabbit antibody, Rockland, Limerick, USA). Flow cytometry was performed with Beckman Coulter Summit v. 4.3.02 software using CyAn ADP analyser machine.

1. **ELISA manufacturer details**

CXCL12 (Cat # 350, R&D systems, Abingdon, UK),

CCL2 (Cat # DY279, R&D systems, Abingdon, UK),

CCL18 (Cat # DY394, R&D systems, Abingdon, UK),

Interleukin 12 (IL-12) (Cat # DY1270, R&D systems, Abingdon, UK),

Interferon Gamma (IFN-γ) (Cat # DY285, R&D systems, Abingdon, UK),

Transforming Growth Factor Beta 1 (TGF-β1) (Cat # DY240, R&D systems, Abingdon, UK)

1. Statistical

The data were expressed as mean +/- standard error of mean. The distribution of the data were analysed with a Kolmogorov-Smirnov test, and a Shapiro Wilk normality test. The statistical significance of differences among three or more variables for data with normal distribution was assessed by one way analysis of variance (ANOVA) with post hoc analysis by Bonferroni, and we used Kruskal–Wallis analysis of variance, with post hoc analysis by Dunn’s test for the data which was not normally distributed. The t test was used for analysing 2 groups with normal distribution and Mann-Whitney test for non-parametric distribution.

**Inclusion criteria for the identification and recruitment of study subjects**

|  | ***H-NS (9)**  **Control** | **†H-S (8)**  **(Control)** | **‡COPD-S (10)** | **§IPF-NS (7)** | **\|\|IPF-S (4)** |
| --- | --- | --- | --- | --- | --- |
| **Gender** | Male/  Female | Male/  Female | Male/ Female | Male/  Female | Male/ Female |
| **Age** | ≧ 50 yrs | ≧ 50 yrs | ≧ 50 yrs | ≧ 50 yrs | ≧ 50 yrs |
| **Able to provide consent** | Yes | Yes | Yes | Yes | Yes |
| **Urinary tobacco metabolites** | Not detected | Detected | Detected | Not detected | Detected |
| **Diagnosis of IPF based on clinical, radiologic +/- histopathologic examination, following multidisciplinary consensus from consultant respiratory physician, consultant radiologist +/- consultant histopathologist.** | No Evidence | No Evidence | No Evidence | Evident | Evident |
| **COPD as defined by the **GOLD criteria, i.e. have spirometry showing**  **†† FEV_1_/FVC <0.70.** | No  Evidence | No  Evidence | Evident | No Evidence | No Evidence |

* Healthy Non Smokers, **†** Healthy Smokers, **‡** Chronic Obstructive Pulmonary Disease – Smokers, **§** Idiopathic Pulmonary Fibrosis – Non Smokers, **||** Idiopathic Pulmonary Fibrosis – Smokers,

****** GOLD Global Initiative for Chronic Obstructive Lung Disease

**††** Pulmonary function testing parameters; FEV_1_ - forced expiratory volume in 1 sec

FVC - forced vital capacity

**Exclusion criteria for the identification and recruitment of study subjects**

| 1. Age less than 50 years 2. Unable to provide consent 3. Major depression or any other significant psychiatric disorder. 4. Illicit drug use within the past six months. 5. Cardiovascular, cerebrovascular, peripheral, and pulmonary vascular disease. 6. Moderate to severe chronic kidney disease (eGFR <30ml/min/1.78m^2^). 7. Severe liver disease (serum Alanine Transferase level >150IU/L). 8. Chronic cystitis, collagen vascular diseases, and skin disorders. 9. Chronic pancreatitis / recurrent acute pancreatitis. 10. Asthma, fibrosis other than IPF or COPD and active neoplasia. 11. Active infection in the preceding 2 weeks. 12. Subjects taking any medication, prescribed or otherwise, that in the opinion of the investigator would preclude the subject from entering the study. 13. Any clinically significant chronic disease that might, in the opinion of the investigator, interfere with the evaluations. |
| --- |
